# Identification of New Potential Interaction Partners for Human Cytoplasmic Copper Chaperone Atox1: Roles in Gene Regulation?

**DOI:** 10.3390/ijms160816728

**Published:** 2015-07-23

**Authors:** Helena Öhrvik, Pernilla Wittung-Stafshede

**Affiliations:** 1 Department of Medical Biochemistry and Microbiology, Uppsala University, Uppsala 751 23, Sweden; E-Mail: helena.ohrvik@imbim.uu.se; 2 Department of Chemistry, Umeå University, Umeå 901 87, Sweden

**Keywords:** copper chaperone, Atox1, transcription factor, two-hybrid screen, bioinformatics

## Abstract

The human copper (Cu) chaperone Atox1 delivers Cu to P_1B_ type ATPases in the Golgi network, for incorporation into essential Cu-dependent enzymes. Atox1 homologs are found in most organisms; it is a 68-residue ferredoxin-fold protein that binds Cu in a conserved surface-exposed Cys-X-X-Cys (CXXC) motif. In addition to its well-documented cytoplasmic chaperone function, in 2008 Atox1 was suggested to have functionality in the nucleus. To identify new interactions partners of Atox1, we performed a yeast two-hybrid screen with a large human placenta library of cDNA fragments using Atox1 as bait. Among 98 million fragments investigated, 25 proteins were found to be confident interaction partners. Nine of these were uncharacterized proteins, and the remaining 16 proteins were analyzed by bioinformatics with respect to cell localization, tissue distribution, function, sequence motifs, three-dimensional structures and interaction networks. Several of the hits were eukaryotic-specific proteins interacting with DNA or RNA implying that Atox1 may act as a modulator of gene regulation. Notably, because many of the identified proteins contain CXXC motifs, similarly to the Cu transport reactions, interactions between these and Atox1 may be mediated by Cu.

## 1. Introduction

Cu is found in the active sites of essential proteins that participate in cellular reactions such as respiration, antioxidant defense, neurotransmitter biosynthesis, connective-tissue biosynthesis and pigment formation [1,2,3]. The redox ability (switch between Cu^+^ and Cu^2+^) of Cu allows Cu-containing proteins to play important roles as electron carriers and redox catalysts in living systems. To avoid toxicity of Cu^+^, the intracellular concentration of Cu is regulated via dedicated proteins that facilitate its uptake, efflux, as well as distribution to target Cu-dependent proteins and enzymes [4,5,6]. In humans, the 68-residue antioxidant 1 copper chaperone (Atox1) picks up Cu that has entered the cell via the membrane-bound Cu importer Ctr1 and delivers the metal to cytoplasmic metal-binding domains in ATP7A and ATP7B (also called Menke’s and Wilson disease proteins, respectively), two homologous multi-domain P_1B_-type ATPases located in the trans-Golgi network. Many human copper-dependent enzymes acquire Cu from these ATPases before reaching their final destination in the body [4,5,6].

Atox1 binds Cu via two conserved Cys residues in a surface-exposed MTCXXC (X, any residue) copper-binding motif [1,7]. Intriguingly, Atox1 contains an apparent nuclear localization sequence (NLS) KKTGK within its C-terminal part and, although not discussed, in the initial discovery paper of Atox1 from 1999 [8], immunofluorescence of HeLa cells indicated that Atox1 was distributed throughout the cell, including the nucleus. Although Atox1 is monomeric in solution, it forms Cu-dependent hetero-dimers with target domains in ATP7A/B that are structurally similar to the homo-dimer of Atox1 found in the X-ray structure [9,10,11,12,13,14,15]. In these dimers, a Cu ion bridges two proteins via interactions with cysteine sulfurs from both metal-binding sites [16].

In 2008, it was reported that Atox1 had an additional activity: Acting as a Cu-dependent transcription factor (TF) that drives the expression of *Ccnd1* (cyclin D1), a protein involved in cell proliferation. Increased levels of cyclin D1 together with other factors promote the transition from G1 to S phase in the cell cycle. To induce and maintain increased expression of cyclin D1, the growth factor signal ERK has to be activated [17]. A direct Cu-dependent interaction of Glutathione *S*-transferase-tagged Atox1 with a GAAAGA sequence in the promotor region of the *Ccnd1* gene was demonstrated by an electrophoretic mobility shift assay [18]. The GAAAGA sequence was also identified in the promoter region of the gene for superoxide dismutase 3 (SOD3), which is the major extracellular antioxidant protein in humans [19,20]. Independently it was demonstrated that Atox1 expression and nuclear accumulation were stimulated by cell treatment with the cancer drug cisplatin [21]. We recently confirmed the presence of Atox1 in the nucleus of HeLa cells but no DNA binding of Atox1 to the proposed GAAAGA promotor sequence *in vitro* was detected [22]. Taken together, the data clearly points to a role for Atox1 in the nucleus, but the specific interaction partners and activities remain unresolved.

Two-hybrid screens using the protein of interest as bait, often in an yeast system, is a powerful tool and highly utilized method to identify known and unknown protein-protein interactions and, thus, assign new functions of proteins [23]. We here used a yeast two-hybrid screen from Hybrigenics to unbiased and systematically identify novel interaction partners of the human Atox1. The same screening approach was used to define an elaborate protein-protein interaction map of the human gastric pathogen *Helicobacter pylori* which allowed connection of 47% of the proteome [23]. Using Atox1 as the bait, we screened 98 million human protein fragments and identified confident hits that corresponded to 25 unique proteins. To differentiate which of the 16 characterized protein targets may be of highest biological relevance as Atox1 interaction partners in the nucleus, a set of bioinformatics analyses were executed. Our investigation provides predictions of new functional roles for Atox1 and identifies what target proteins to prioritize in follow-up biochemical studies to further explore the functional role of Atox1 in the nucleus.

## 2. Results and Discussion

### 2.1. Atox1 Interaction Partners from Two-Hybrid Screen

To search for new interaction partners of Atox1 in an unbiased way, we employed a well-established yeast two-hybrid screen using Atox1 as the bait. Upon screening a human placenta library of cDNA fragments, we tested over 98 million interactions that are 9-fold more than the library complexity. The human placenta library was selected as it is a generic library that expresses a large variety of transcripts. Three hundred and ten positive clones were fully processed (*i.e.*, did not fail to grow and could be sequenced). In initials tests, the bait fusion of Atox1 was not toxic or auto-activating the yeast two-hybrid system. Only a low (0.5 mM) 3-aminotriazole concentration was required for the spread of the mating to increase the selection stringency. Since Cu may be involved in and regulate Atox1 activities, as well as increase the nucleus accumulation of Atox1, 10 µM Cu was added to the selective medium.

**Figure 1:**
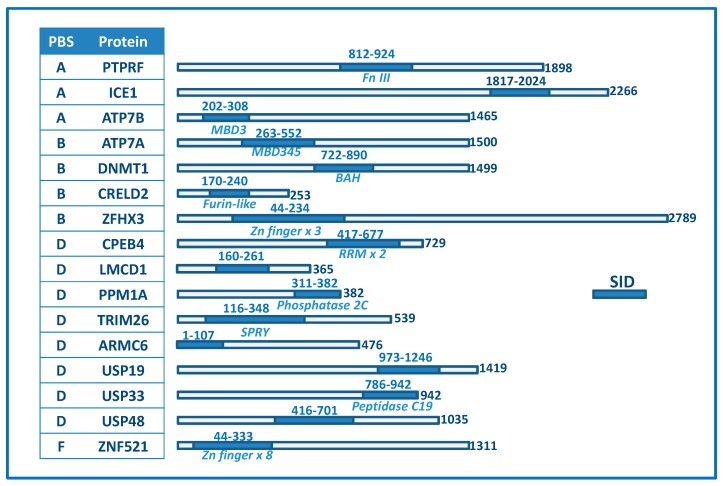
Predicted Biological Score (PBS) score, name, and SID domain boundaries for Atox1 identified interaction partners. The length of each protein is given and, if the SID overlaps with a known domain, this is listed in italics under the SID. Only SIDs are annotated in terms of known domains but for many of the proteins additional domains are predicted/known. Fn III, fibronectin domain 3; MBD, metal-binding domain; BAH, bromo-adjacent domain; Zn, zinc.

Several preys were found with high confidence (Predicted Biological Score (PBS) of A or B) which suggested that the bait was well folded. Because the screen was technically successful, the PBS D preys (there were no C prey) were also considered confident interaction partners. In summary, 25 prey proteins were identified with high confidence (PBS A–F; there were no E prey but one F prey and we kept it for analysis). Of the 25 selected proteins, 9 were uncharacterized proteins. The remaining 16 proteins were pursued for bioinformatics and sequence/structure analyses (Table S1, Figure 1). Because the screen contains several fragments (~9) of each prey protein, it was possible to identify what segment/domain (termed selected interaction domain, SID) in each prey protein was responsible for the Atox1 interaction. Because interactions with the isolated SID domains were observed, it points to these protein fragments being well folded structures. If the identified SID segment corresponded to an identified domain, this is listed in Table S1 and Figure 1.

### 2.2. Detection of Two “Positive Controls”

As expected, we found both ATP7A and ATP7B among the identified high confidence hits. Because they are established partners of Atox1 in the cytoplasmic Cu transport pathway we consider them as positive controls. To note is the observation that in ATP7A, the interacting domains are metal-binding domains 3, 4 and 5, whereas in ATP7B, the interacting domain is metal-binding domain 3 only (Figure 1). This represents the metal-binding domains in each case that form the most stable (in contrast to transient) interactions with Atox1, and/or it implies that, in a cellular setting, these are the selected domains to which Atox1 delivers Cu. Although *in vitro* studies have shown that Atox1 can deliver Cu to all six domains in both ATP7A and ATP7B and, in the case of ATP7B, stable Cu-mediated Atox1 hetero-complexes are formed only with metal-binding domains 1, 2 and 4 [24], the screen results imply that the scenario *in vivo* may be different. This will be important to explore in future studies.

### 2.3. Bioinformatics Analysis of Original Hits

To obtain information of the remaining 14 confident hits, we utilized the power of bioinformatics. In Table S1, the annotated function (GeneOntology) for each protein is given and, in Table S2, protein localization and tissue distribution information taken from the human protein atlas (www.proteinatlas.org/) is reported. Among the 14 proteins, the following six were reported as detected in the nucleus: ICE1, DNMT1, ZHFX3, TRIM26, USP48, and ZNF521 (Table S2). Several of the nuclear proteins are known DNA/RNA-binding proteins (ICE1, DNMT1, ZHFX3, TRIM26, and ZNF521) and this activity was reported also for CPEB4, LMCD1 and PPM1A although nuclear localization had not been confirmed (Table S1).

**Figure 2:**
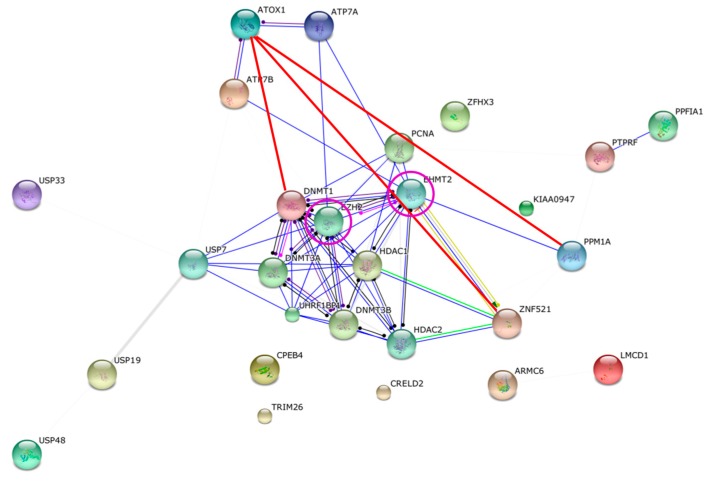
Predicted interaction network for Atox1 and the confident hits based on String analysis (all arrows except the red lines). Atox1 interacts with ATP7A/B, as found in the current screen and in its normal Cu transport function and also predicted by String. Three Atox1 interaction partners from the screen (red lines; ZNF521, PPM1A, DNMT1) as well as ATP7A/B were proposed by String to interact with the proteins EZH2 and/or EHMT2 (purple circles). These interactions create closed networks linking Atox1 to the three screen hits also via the ATP7A/B and EZH2/EHMT2 connections. None of the other confident Atox1-interacting hits demonstrated any connectivity within this set of proteins although the confident level was set to low.

To investigate known and predicted protein interactions for the confident hits, to assess if they are linked with each other, we turned to the STRING database (String-db.org). This database provides direct (physical) and indirect (functional) associations based on quantitative integration of interaction data from four sources (genomic context, high-throughput experiments, co-expression data and previous knowledge) for a large number of organisms and transfers information between organisms where applicable. Using the lowest confidence scores and all the confident hits, the String output confirmed the Atox1 interaction partners ATP7A and ATP7B and unexpectedly predicted that they interact with proteins named EHMT2 and EZH2. These two proteins are not among the discovered hits but they exhibit direct interactions with three proteins on our list: DNMT1, ZNF521 and PPM1A. This implies closed-circle protein-protein interactions networks with EHMT2 and EZH2 connecting ATP7A/B to some of the Atox1 interacting hits (Figure 2).

Sequence analysis (Meme-suite.org) of the hits revealed two sequence motifs that occurred in many of the hit proteins: **Tyr-**X**-Cys-Glu**-X-**Cys** and **Gly-Leu-X**-**Asn-**X**-Gly-**X-**Thr-Cys-**X-X-**Asn-**X-X-X-**Gln** (X, any residue), where the first motif agrees with many of the proteins having C_2_H_2_ zinc fingers in some of their domains. Notably, only two of the SID fragments interacting with Atox1 contained Cys_2_ motifs (the SID fragments from ZFHX3 and ZNF521). We note that in these two cases, one must consider the Cu-Atox1 interaction as a possible artifact driven by Cu-mediated connection of two CXXC motifs.

### 2.4. Differentiating among the Discovered Interaction Partners

**Figure 3:**
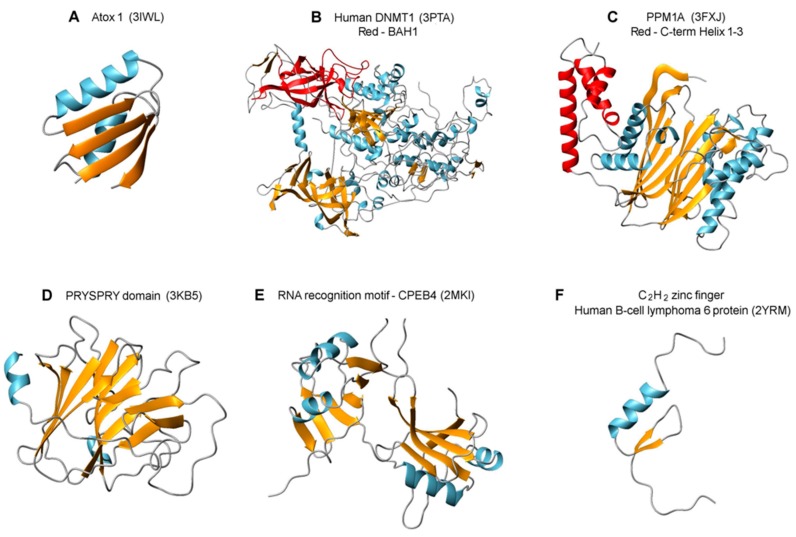
Structures of interacting proteins or protein domains taken from the protein data bank. (**A**) Atox1, the bait; (**B**) DNMT1 with the first BAH domain (interaction partner to Atox1) in red; (**C**) PPM1A with the three C-terminal helices (interacting with Atox1) in red. (**D**) PRYSPRY domain of TRIM72 (matching the same domain of TRIM26 with which Atox1 interacts); (**E**) The RNA recognition motif (RRM) of CPEB4; and (**F**) A zinc finger domain, as found in several of the hit proteins.

To create a priority list among the discovered Atox1 interactions partners, we scored (1/0) the proteins based on four parameters (Table 1). To get a score of 1 in each category, the PBS score should be A or B (and not D or E), the protein should be localized in the nucleus, the protein should interact with nucleic acids, and there should be a predicted network connection to Atox1 (via other proteins). Based on this scoring, the following seven proteins appear as most interesting to investigate further (top scores of 4, 3 and 2): DNMT1, ICE1, ZFHX3, CPEB4; PPM1A, TRIM26 and ZNF521. Inspection of Figure 1 demonstrates that the SID domains (*i.e.*, the segment in each protein to which Atox1 was found to interact) correspond to known structural domains for all these proteins but ICE1. In Figure 3, we show structures of these proteins and/or relevant interaction domains (the BAH (bromo-adjacent homology) domain in DNMT1, the tandem RNA recognition motif in CPEB4, a PRYSPRY domain as found in TRIM26, PPM1A, and a typical C_2_H_2_ zinc-finger domain) together with the structure of Atox1. Most of these are “new”, eukaryote-specific proteins that have many interactions partners; some of their key functional/structural features are discussed below. We note that the other hits may also be of high importance and each one should be carefully investigated. For example, it was recently discovered that LMCD1 could be localized in the nucleus and point-mutated variants acted as a putative metastatic oncogene in human hepatocellular carcinoma [25]. Because the liver has an elaborate Cu transport system, Atox1 is highly expressed in this organ.

**Table 1:** Scoring of protein hits. The 14 novel target proteins were scored (1/0) according to the following criteria: nuclear localization, nucleic acid binding, network connection, PBS score. A score of 1 was assigned if the protein is found in the nucleus, if it binds nucleic acids, if it is connected to Atox1 via network, and if the PBS score was A or B.

Target Protein	Found in Nucleus	DNA/RNA Binding	String Network	High PBS	Total
PTPRF	0	0	0	1	1
ICE1	1	1	0	1	3
DNMT1	1	1	1	1	4
CRELD2	0	0	0	1	1
ZFHX3	1	1	0	1	3
CPEB4	1	1	0	0	2
LMCD1	0	1	0	0	1
PPM1A	0	1	1	0	2
TRIM26	1	1	0	0	2
ARMC6	0	0	0	0	0
USP19	0	0	0	0	0
USP33	0	0	0	0	0
USP48	1	0	0	0	1
ZNF521	1	1	1	0	3

### 2.5. Features of Top Targets Hinting at New Roles for Atox1

Methylation of cytosine in DNA plays a crucial role in development through inheritable gene silencing. The DNA methyltransferase DNMT1 is responsible for the propagation of methylation patterns to the next generation via its preferential methylation of hemi-methylated CpG sites in the genome [26]. Thus, like cyclin D1 (whose expression was proposed to be driven by Atox1, as mentioned in the introduction) DNMT1 expression is cell cycle dependent and, in fact, the expression patterns of DNMT1 and cyclin D1 during the cell cycle are known to correlate [27]. In DNMT1, Atox1 interacted with the first BAH domain which has β-structure with many surface exposed regions serving as a platform for protein-protein interactions. Notably there is a proximal C_3_H zinc-binding site in the connecting linker between the CXXC domain that interacts with DNA and the first BAH domain. Since DNMT1 is thought to be in an auto-inhibitory state in which the linker from the CXXC domain stretches over the first BAH domain and blocks DNA from entering the catalytic site [28], excitingly, one may speculate that protein (*i.e.*, Atox1) binding to the first BAH domain may turn on activity.

The tandem RNA recognition motif (RRM) that Atox1 interacts with in CPEB4 is one of the most abundant protein domains in eukaryotes and consists of a 4-stranded β-sheet and 2 α-helices in each motif [29]. CPEB proteins bind cytoplasmic polyadenylation elements of mRNA (elements involved in regulation of cell proliferation, chromosome segregation and cell differentiation) via its RRM and can act as either translational repressors or activators. CPEB4 was observed to support tumor growth, via translational activation of mRNAs that are silenced in normal tissue, in pancreatic tumors [30]. Reported protein interactions with RRMs are diverse and have been shown to involve (1) acting as cofactors mediating RNA binding; (2) binding the RRM β-sheet preventing RNA interactions; and (3) interacting with the helices tuning RRM binding to RNA [31]; thus, Atox1 interaction with CPEB4 may result in post-transcriptional regulation in many ways. Notably, cyclin D1 expression is linked to the expression of CPEB4 through the activation of ERK and it was also discovered that down-regulation of CPEB4 promoted G1 to S phase transition [17]. Thus, an Atox1 interaction with CPEB4 (as implied from the two-hybrid screen here) that is inhibitory may result in increased levels of cyclin D1 and promotion of cell proliferation, as reported previously [18].

The Atox1 interaction partners TRIM26 and PPM1A are both involved in viral defense signaling and stress response. In resemblance, Atox1 was originally discovered as an antioxidant protein expressed in response to oxidative stress and current considerations involve possible roles in cell redox sensing. The proteins in the TRIM family have different functions related to immune response and viral growth restriction; they are often expressed in response to interferons. In TRIM26, Atox1 was found to interact with the C-terminal part containing a SPRY domain, that is evolutionary ancient, and a preceding PRY domain, only found in vertebrates (so-called PRYSPRY motif) [32]. The PRYSPRY domains may function as hetero-dimers that involve β-strand swapping between two TRIM monomers, which results in the formation of putative interaction sites able to recognize a discrete range of target proteins (possibly including Atox1), similarly to antibody-antigen interactions [32]. PPM1A (also called PP2CA) is an essential manganese/magnesium ion-dependent Ser/Thr phosphatase that regulates cellular stress response in eukaryotes [33]. Specifically, it was shown to negatively regulate antiviral signaling by catalyzing de-phosphorylation of the immune-system related proteins STING and TBK1 [34]. Reversible phosphorylation of proteins acts as activation/inactivation mechanisms in many cellular processes (including cell cycle progression). Based on the two-hybrid screen result, Atox1 interacted with the C-terminal helices in PPM1A, which are unique to mammalian PP2C isoforms [35] and are thought to contribute to substrate specificity. Notably, Atox1 is predicted to be phosphorylated at Ser44 and Ser47 (http://www.phosphosite.org) and if so, in order to be recycled, there may be a requirement for a phosphatase such as PPM1A during the functional cycle of Atox1 and encountered cellular stress.

Finally, both ZFHX3 and ZNF521 proteins contain a number of zinc-finger (ZF) domains that are the most common DNA-binding motifs found in eukaryotic transcription factors. In ZFHX3, the SID domain for Atox1 involved a stretch of three C_2_H_2_-ZF domains of the U1-type whereas in ZNF521, the SID for Atox1 covered at least 7 or 8 classical C_2_H_2_-ZF domains (Figure 1). ZF motifs are stable scaffolds found in many proteins with diverse functions; in addition to DNA binding, ZF motifs can also bind to RNA and protein targets [36]. Although ZFHX3 and ZNF521 are poorly characterized proteins, they are implied in transcription activity and nucleic acid binding, respectively (Table S1). Thus, if Atox1 interacts with some of their DNA-binding motifs, as predicted, these interactions may clearly have potency to turn on or off gene expression.

## 3. Materials and Methods

### 3.1. Yeast Two-Hybrid Analysis

Yeast two-hybrid screening was performed by Hybrigenics Services, Paris, France (http://www.hybrigenics-services.com). The coding sequence for homo sapiens Atox1 (1-68) (GenBank accession number gi: 72004264) was PCR-amplified and cloned into vector pB27 as a C-terminal fusion to LexA (N-LexA-ATOX1-C). The construct was checked by sequencing the entire insert and used as a bait to screen a random-primed Human Placenta cDNA library constructed into vector pP6. pB27 and pP6 derive from the original pBTM116 [37] and pGADGH plasmids, respectively. 98 million clones (9-fold the complexity of the library) were screened using a mating approach with YHGX13 (Y187 ade2-101::loxP-kanMX-loxP, matα) and L40ΔGal4 (mata) yeast strains (with *His3* as a selection gene) as previously described [38]. 310 His+ colonies were selected on a medium lacking tryptophan, leucine and histidine, and supplemented with 0.5 mM 3-aminotriazole to handle bait autoactivation and 10 μM CuSO_4_. The prey fragments of the positive clones were amplified by PCR and sequenced at their 5′ and 3′ junctions. The resulting sequences were used to identify the corresponding interacting proteins in the GenBank database (NCBI) using a fully automated procedure. A confidence score (PBS, for Predicted Biological Score) was attributed to each interaction as previously described [39].

### 3.2. Confidence Scores

The PBS relies on two different levels of analysis. Firstly, a local score takes into account the redundancy and independency of prey fragments, as well as the distribution of reading frames and stop codons in overlapping fragments; Secondly, a global score takes into account the interactions found in all the screens performed at Hybrigenics using the same library. This global score represents the probability of an interaction being nonspecific. For practical use, the scores were divided into four categories, from A (highest confidence) to D (lowest confidence). A fifth category (E) specifically flags interactions involving highly connected prey domains previously found several times in screens performed on libraries derived from the same organism; Finally, several of these highly connected domains have been confirmed as false-positives of the technique and are now tagged as F. The PBS scores have been shown to positively correlate with the biological significance of interactions [23,40].

### 3.3. Bioinformatics

The GeneID for all confident hit proteins found in the screen are listed in Table S3. Websites for bioinformatics analyses are given in the text.

## 4. Conclusions

Because Atox1 was proposed to have functionality outside of the cytoplasm [18,19,20], and we as well as others have detected it in the nucleus [21,22], we were captivated to explore the cellular interaction network for Atox1. The results of the yeast two-hybrid screen presented here suggest that Atox1 have several novel interaction partners not related to cytoplasmic Cu transport. Some of these new interactions may explain the presence of Atox1 in the nucleus and its proposed role in cell proliferation whereas others may uncover new, as yet unknown functions. Several of the identified target proteins are involved in DNA/RNA binding which places Atox1 as a putative gene modulator. Because many of the identified interacting proteins are eukaryotic-specific, it is conceivable that additional functionalities for Atox1 have evolved during evolution in order to adapt to increased organism complexity.

Five of seven nucleic acid interacting proteins identified in our yeast two-hybrid screen bind metals suggesting that Atox1’s Cu-binding ability may play a role in the interactions. In addition to Cu being an essential cofactor in proteins, a range of regulatory roles of Cu have emerged in recent years [41]. One may speculate that some of these regulatory effects are mediated via Cu-bound Atox1 in the nucleus through the identified proteins. Because of the potential importance of Cu binding to Atox1, a crucial follow up will be to elucidate the regulatory effect of Cu-binding to Atox1 by performing interaction experiments without Cu added to the media. Although the predictions from the two-hybrid screen results will require extensive biochemical follow up with purified proteins, in both the presence and absence of Cu, this study identifies possible interaction partners for Atox1 in the nucleus.

## Supplementary Materials

Supplementary materials can be found at http://www.mdpi.com/1422-0067/16/08/16728/s1.
