# Supplementary material for: Identification of New Potential Interaction Partners for Human Cytoplasmic Copper Chaperone Atox1: Roles in Gene Regulation?
Source: Int J Mol Sci. 2015 Jul 23;16(8):16728–39. doi: 10.3390/ijms160816728 (PMC4581165; doi:10.3390/ijms160816728)
Supplement: Supplementary file 1 [file ijms-16-16728-s001.pdf]

Supplementary Information

Table S1. Confident hits from a cDNA yeast two-hybrid screen using Atox1 as bait and a human placenta RP6 fragment library as prey. Detected interactions with highest predicted biological scores (PBS) are listed below divided in categories from A (highest confidence rank) to F. No C or E scores were found. Functional annotation extracted from Geneontology. (Nine additional hits were detected that corresponded to Genbank IDs of unknown functions, not listed.)

PBS	Target	Function (Direct Annotation)
A	PTPRF	Cell migration; transmembrane receptor protein tyrosine phosphatase activity; integral component of plasma membrane; cell adhesion; negative regulation of receptor binding; peptidyl-tyrosine dephosphorylation
A	ICE1	snRNA transcription from RNA pol III promoter; positive regulation of protein complex assembly; transcriptionally active chromatin; positive regulation of intracellular protein transport; transcription elongation factor complex
A	ATP7B	Known Atox1 partner (positive control), Cu binding
B	ATP7A	Known Atox1 partner (positive control), Cu binding
B	DNMT1	DNA methylation on cytosine; DNA-methyltransferase activity; regulation of gene expression, epigenetic *zinc binding*
B	CRELD2	Cysteine-rich with EGF-like domain protein 2; protein binding; calcium binding
B	ZFHX3	Protein binding; sequence-specific DNA binding transcription factor activity *zinc binding*
D	CPEB4	Cytoplasmic polyadenylation element-binding protein 4 nucleotide binding; nucleic acid binding; RNA binding
D	LMCD1	Negative regulation of transcription; transcription corepressor; zinc binding
D	PPM1A	Insulin receptor signaling pathway; transcription initiation from RNA pol II promoter; growth factor beta receptor pathway; protein dephosphorylation; protein ser/thr phosphatase; manganese and magnesium binding
D	TRIM26	Negative regulation of viral release from host cell; positive regulation of sequence-specific DNA binding; transcription factor; innate immune response; zinc binding
D	ARMC6	hematopoietic progenitor cell differentiation
D	USP19	cysteine-type endopeptidase activity; protein de-ubiquitination
D	USP33	cysteine-type endopeptidase activity; protein de-ubiquitination
D	USP48	cysteine-type endopeptidase activity; protein de-ubiquitination
F	ZNF521	nucleic acid binding; metal ion binding

Table S2. Tissue and cellular localization data for Atox1 (bait) and the 14 high-scoring hits (established partners ATP7A/B not included here) taken from ProteinAtlas.

Target	Tissue Distribution	Cellular Localization
Atox1(bait)	Hepatocytes, renal tubules and seminal vesicle displayed strong cytoplasmic positivity. Lower stomach, thyroid gland, intestinal tract and subset of cells in seminiferus ducts of testis were moderately stained. Remaining normal cells were negative.	Mainly localized to the nucleus but excluded from the nucleoli. In addition localized to the plasma membrane in human cells. Localized to the cytoplasm in mouse cells.
PTPRF	Most normal tissues showed moderate to strong cytoplasmic staining. Cells in CNS, liver, squamous epithelium, smooth and skeletal muscle were weakly stained or negative.	Localized to the Golgi apparatus.
ICE1	Most of the normal cells displayed weak to moderate nuclear positivity with additional cytoplasmic and/or membranous staining in several cases. Fallopian tube and pre-menopausal were strongly stained. Glial cells, prostate and soft tissues were negative.	Mainly localized to the actin filaments. In addition localized to the nucleus but excluded from the nucleoli.
DNMT1	Distinct nuclear expression in dividing cells (blood and immune system, placenta, testis).	Localized to the nucleus but excluded from the nucleoli.
CRELD2	Cytoplasmic expression in a majority of tissues. High expression in exocrine pancreas.	N.D.
ZFHX3	Most normal tissues showed weak to moderate nuclear positivity. Gastrointestinal tact, thyroid, urothelium and superficial layer in squamous epithelia exhibited strong nuclear and cytoplasmic immunoreactivity. Liver, lung, breast, glial cells, lymphoid tissues and soft tissues were negative.	Mainly localized to the nucleus (+vesicles).
CPEB4	Cytoplasmic expression in most cells.	Localized to the Golgi apparatus, vesicles & nucleus but excluded from the nucleoli.
LMCD1	Most of the normal tissues exhibited weak to moderate cytoplasmic and/or membranous positivity. Strong cytoplasmic staining was observed in Purkinje and molecular layer cells in cerebellum. Hepatocytes, pancreatic islet cells, glial cells and lymphoid tissues were generally negative.	Mainly localized to the plasma membrane. In addition localized to the cytoplasm & cell junctions.
PPM1A	Normal tissues displayed weak to moderate cytoplasmic staining. Membranous staining was observed in glandular cells of cervix, prostate and stomach. Hematopoietic cells, small intestine and renal tubules were strongly stained.	N.D.
TRIM26	Ubiquitous cytoplasmic expression.	Mainly localized to the nucleus but excluded from the nucleoli. In addition localized to the cytoplasm.
ARMC6	Cytoplasmic expression in several tissues.	Cytoplasm.
USP19	N.D.	N.D.
USP33	Most of the normal tissues displayed moderate cytoplasmic positivity. Alveolar cells, liver and lymphoid tissues were weakly stained or negative.	Cytoskeleton (Intermediate filaments).
USP48	Ubiquitous cytoplasmic expression.	Mainly localized to the nucleus but excluded from the nucleoli. In addition localized to the cytoplasm & mitochondria.
ZNF521	General nuclear expression which in many cells was combined with weaker cytoplasmic expression.	Nucleus.

N.D. = not determined.

Table S3. Gene ID numbers and names for hit proteins.

Gene ID Number	Names for Hit Proteins
PTPRF	|gi|109633039 receptor-type tyrosine-protein phosphatase F isoform 2 precursor
ICE1	|gi|149363685 little elongation complex subunit 1
ATP7A	|gi|532691750 copper-transporting ATPase 1 isoform 1
ATP7B	|gi|55743071| copper-transporting ATPase 2 isoform a
DNMT1	|gi|195927037 DNA (cytosine-5)-methyltransferase 1 isoform a
CRELD2	|gi|205360958 cysteine-rich with EGF-like domain protein 2 isoform b precursor
ZNFHX3	|gi|258613987 zinc finger homeobox protein 3 isoform B
CPEB4	|gi|189027090 cytoplasmic polyadenylation element-binding protein 4
LMCD	|gi|7657309 LIM and cysteine-rich domains protein 1 isoform 1
PPM1A	|gi|10337595 protein phosphatase 1A isoform 1
TRIM26	|gi|4508005 tripartite motif-containing protein 26
ARMC6	|gi|15529984 armadillo repeat-containing protein 6 isoform 2
USP19	|gi|312596871 ubiquitin carboxyl-terminal hydrolase 19 isoform
USP33	|gi|42516567 ubiquitin carboxyl-terminal hydrolase 33 isoform
USP48	|gi|52630449 ubiquitin carboxyl-terminal hydrolase 48 isoform a
ZNF521	|gi|24308069 zinc finger protein 521
